# Digital stereophotogrammetry based on circular markers and zooming cameras: evaluation of a method for 3D analysis of small motions in orthopaedic research

**DOI:** 10.1186/1475-925X-10-12

**Published:** 2011-02-01

**Authors:** Evgenij Bobrowitsch, Christof Hurschler, Gavin Olender, Christian Plaass, Hazibullah Waizy, Heino Arnold, Christina Stukenborg-Colsman

**Affiliations:** 1 Laboratory for Biomechanics and Biomaterials; Department of Orthopaedic Surgery; Hannover Medical School, Hannover, Germany; 2 Department of Orthopaedic Surgery; Hannover Medical School, Hannover, Germany; 3 Orthopädisch - Unfallchirurgische Praxisklinik Rehau; Orthopädisches Zentrum Fichtelgebirge am Klinikum, Germany

## Abstract

**Background:**

Orthopaedic research projects focusing on small displacements in a small measurement volume require a radiation free, three dimensional motion analysis system. A stereophotogrammetrical motion analysis system can track wireless, small, light-weight markers attached to the objects. Thereby the disturbance of the measured objects through the marker tracking can be kept at minimum. The purpose of this study was to develop and evaluate a non-position fixed compact motion analysis system configured for a small measurement volume and able to zoom while tracking small round flat markers in respect to a fiducial marker which was used for the camera pose estimation.

**Methods:**

The system consisted of two web cameras and the fiducial marker placed in front of them. The markers to track were black circles on a white background. The algorithm to detect a centre of the projected circle on the image plane was described and applied. In order to evaluate the accuracy (mean measurement error) and precision (standard deviation of the measurement error) of the optical measurement system, two experiments were performed: 1) inter-marker distance measurement and 2) marker displacement measurement.

**Results:**

The first experiment of the 10 mm distances measurement showed a total accuracy of 0.0086 mm and precision of ± 0.1002 mm. In the second experiment, translations from 0.5 mm to 5 mm were measured with total accuracy of 0.0038 mm and precision of ± 0.0461 mm. The rotations of 2.25° amount were measured with the entire accuracy of 0.058° and the precision was of ± 0.172°.

**Conclusions:**

The description of the non-proprietary measurement device with very good levels of accuracy and precision may provide opportunities for new, cost effective applications of stereophotogrammetrical analysis in musculoskeletal research projects, focusing on kinematics of small displacements in a small measurement volume.

## Background

"Three-dimensional (3D) measurements play a vital role in a diversity of industries and disciplines, ranging from the manufacturing and process sectors to healthcare" [1]. Orthopaedic research often focuses on qualitative and quantitative measurements of an object's motion [2,3]. An image based 3D motion analysis system can track wireless, small, light-weight markers attached to the surface of an object. Measurement of strain on the ligaments or spatial changes of small bones can be determined with a high accuracy. Malicky et al. used a stereoradiogrammetry to measure the strain on the glenohumeral capsule by means of spherical markers [4]. Video-based 3D motion analysis systems based on black [5] or retroreflective [6] spherical markers were developed for tracking objects in small (less than 1 m^3^) measurement volume. In contrast to gait analysis tracking systems configured for large measurement volume [7], "motion analysis configured for registration within small volumes allows measurement of minuscule displacements with great accuracy" [6].

A possibility to optimize the motion system accuracy was successfully tested by Mössner and co-workers [8]. They used zoom, tilt and pan of theirs cameras to track athletes movement during down hill skiing. The zoom was used to overcome the limited camera resolution. The tilt and pan enabled the object tracking in the field of view of the stationary camera while zooming. In order to perform this task, at least 6 control points [9] had to be visible in each camera frame. This required the Direct Linear Transformation (DLT) method [10,11] while determining the intrinsic and extrinsic parameters of a fully projective camera. Due to the known intrinsic parameters, the camera lens distortion can be minimized. This improves the object's spatial information derivated from the captured image. In order to reconstruct 3D positions of markers captured by two or more cameras, the extrinsic parameters of each camera have to be known. These extrinsic parameters describe the geometrical relation between the camera and the captured calibration body. The calibration body is not required during subsequent motion tracking when all cameras remain at the same fixed relative to one another position [5,6]. Otherwise, when experiment conditions require a flexible camera positioning and the camera's intrinsic parameters were determined (the camera was pre-calibrated), we can determine the extrinsic parameters for each camera using at least 3 [12] or 4 [13] control points. This process is also called "camera pose estimation".

Ansar and Daniilidis [13] and Lepetit et al. [14] showed in comparison to different pose estimation methods, a very good accuracy and robustness of the orthogonal iterations algorithm developed by Lu et al. [15]. The orthogonal iterations algorithm searches for an optimal orthogonal projection of the control points presented as a perspective projection on the image plane. The orthogonality constraint is enforced by using singular value decomposition, not from specific parameterization of rotations, e.g., Euler angles [15] typical for DLT methods.

A big calibration body used for the camera calibration in DLT methods can be replaced with a small fiducial marker when the cameras were pre-calibrated. Fiducial markers are artificial landmarks added to a scene to facilitate locating point correspondences between images, or between images and a known model [16]. In our study, the fiducial marker was defined as an aggregate of coplanar control points used for the camera pose estimation. Coplanar reference objects are especially easy to manufacture and measure [17]. In addition, a control point detection based on a detection of the centre of a circular target is advantageous because the circle is centrosymmetric and the detection of the circle centre is not sensitive to the thresholding error [18]. Nevertheless most of 3D tracking systems are based on the use of spherical markers because their circular image is almost independent of the viewing direction [19] of the camera. In contrast to this, the perspective projection of a circle marker on the image-plane has an elliptical form with exception if the image-plane is parallel to the circle-plane. The ellipse centre differs from the centre of the projected circle depending on the angle and displacement between the circle surface and the image-plane. This effect is known as eccentricity [20]. In order to avoid the systematic geometric image measurement error because of the eccentricity, its correction is required [18,20,21].

Using the combination of all advantageous aspects of the different techniques mentioned above, the purpose of this study was to develop and evaluate a non-position fixed motion analysis system configured for a small measurement volume. The system was used zoom to track small round flat markers with respect to the fiducial marker consisting of four coplanar circles. Additionally, a unique solution to find the circle's centre projecting on the image had to be developed and applied. This 3D motion analysis system was specifically configured to measure spatial changes of small bones in the foot region.

## Methods

### Tracking Devise and Image Acquisition

**Figure 1:**
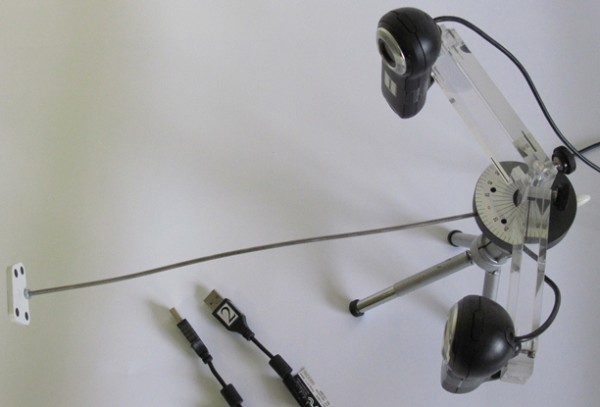
**Stereophotogrammetrical tracking device**. The plastic fiducial marker (left), consisted of four black circles on a white background, was placed on the pin between the two web cameras.

Two web cameras (Logitech^®^, Webcam Pro 9000) were used to acquire static pictures with a resolution of 1600 × 1200 pixels. A camera holder was constructed to allow the camera's viewing axis to cross with an angle of 40° on the object of interest. The plastic fiducial marker consisted of four black circles (ø 5 mm) on a white background and was placed on a 27 cm long pin between the cameras (Figure 1). The black circles on the fiducial marker were mounted with an accuracy of 0.005 mm. The unique arrangement of the four black circles is similar to what was used to build a reference marker in the previous study [22]. The markers to track were single black circles (ø 1 or 2 mm) printed (HP Laserjet 1300, resolution of 1200 × 1200 dpi) on a white background.

Both cameras were pre-calibrated with a fixed focus and camera zoom factor of 2.2 using an internet available tool [23]. A 6.5 × 6.5 cm calibration board was printed by laser printer as a black-white checkerboard presenting a grid of 144 control points. The calibration board was captured inside of a field of view of each camera in 18 different positions fulfilling a volume where the markers to track and the fiducial marker had to be captured. Thus the intrinsic parameters were determined in order to pre-calibrate each camera [23]. Due to the 90° tilted cameras (Figure 1), the measurement volume (approximately 0.1 × 0.1 × 0.1 m) was behind and above the fiducial marker which was placed in the middle of the lower part of each camera field of view.

The image acquisition tool was programmed with MATLAB (The MathWorks Inc., Natick, MA, USA). This tool allowed the real time streaming view from both cameras. When the fiducial marker was placed near to the tracked objects, two static images from both cameras were simultaneously acquired under optimal light conditions (150 Watt, Ministudio 606, Multiblitz Dr. ing. D. A. Mannesmann GMBH & CO KG, Köln, Germany) described in a manual of the used cameras.

### Determination of the circle centre projected onto the image-plane

The black circle on the white background was used to locate the fiducial as well as the object-surface markers. Edge detection between the black and white areas was performed by means of a gray value threshold. On this edge, an ellipse Π^*i*^(*c*^*i*^, *a*^*i*^, *b*^*i*^, *α*^*i*^) was fitted, where *i *was denoted as "initial". The centre *c*^*i *^corresponded only approximately to the centre of the projected circle, when the ellipse axes ratio *a*^*i*^/*b*^*i *^was not equal to one.

**Figure 2:**
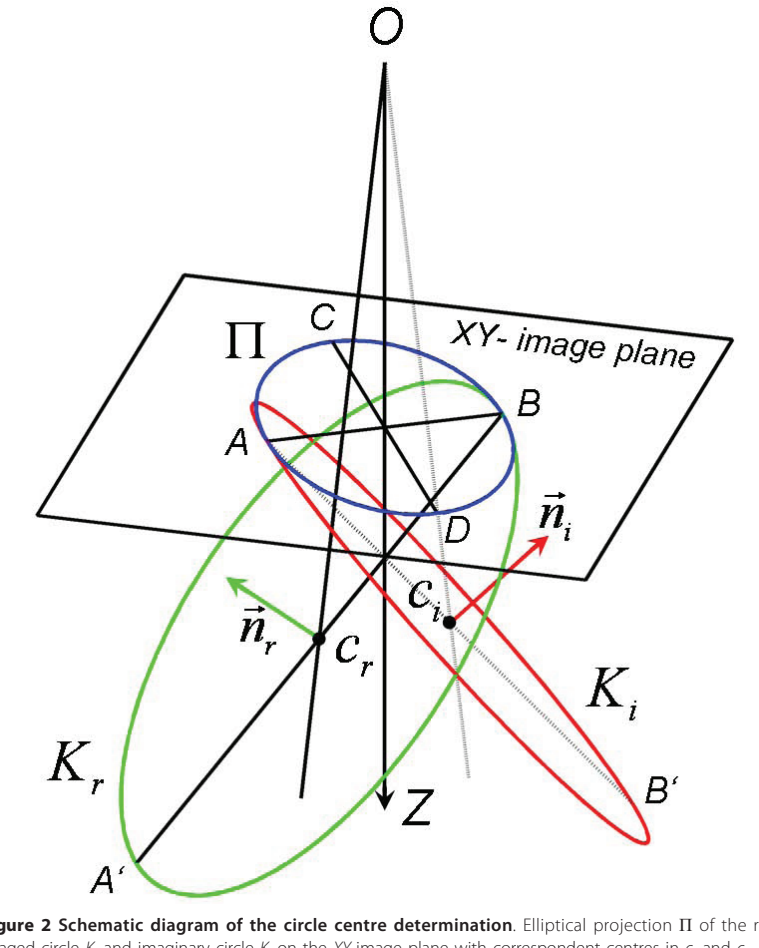
**Schematic diagram of the circle centre determination**. Elliptical projection Π of the real imaged circle *K*_*r *_and imaginary circle *K*_*i *_on the *XY*-image plane with correspondent centres in *c*_*r *_and *c*_*i*_, |*AB*| = 2*a*, |*CD*| = 2*b*, ∠*ABA*' = ∠*BAB*' = *γ*, the distance between the projection centre *O *and the *XY*-plane is equal to *d*. The bisecting line of the cone with the basis Π on the *XY*-plane and the top in the perspective projection centre *O *coincided with the *Z*-axis. See the text for explanation.

When considering the cone with the basis Π^*i *^on the image plane and the top in the perspective projection centre *O*, $\vec{v}$ was defined as the unit vector of the bisecting line of this cone from *O *to Π^*i*^. We rotated the cone about *O *till the cone bisecting line coincided with the image *Z*-axis. The rotation matrix $R(\vec{n},\beta )$ described this rotation, where $\vec{n}$ was the unit vector derived from the cross product of $\vec{v}$ with the image *Z*-axes and *β *was the angle between them. The intersection of the rotated cone with the image *XY*-plane was an ellipse Π(*c,a,b,α*) (Figure 2). If *a *= *b *the ellipse Π was a circle and the normal vector of the circle-plane was the same as the image *Z*-axis. The angle *γ *between the circle-plane and the image *XY*-plane was in this case equal to zero.

(1) $$\text{In the case if}\;a<b\text{ the }\sin\gamma =\pm \sqrt{\left(1-\frac{a^{2}}{b^{2}}\right)/(1+\frac{a^{2}}{d^{2}})},$$

where *d *was the distance from *O *to the image *XY*-plane. The rotation of the minor axis $\vec{a}$ about the ellipse long axis $\vec{b}$ with the amount of ± *γ *yielded two vectors which were useful to find the two sections AB' and BA' whose middle lay on the rays passed through *O *and the centres of the real imaged *c*_*r *_and imaginary *c*_*i *_circles (Figure 2). The reversed rotation with the transposed (*t*) matrix $R^{t}(\vec{n},\beta )$ yielded the searched rays passed trough *O *and the centres of the real imaged and the imaginary circles. The selection criterion for the fiducial marker was that the four real imaged circles were coplanar and their imaginary circles were not coplanar. Furthermore, during reconstruction of the 3D marker position from the left and right images, two normal vectors for each imaged circle were found (${\vec{n}}_{r}$ and ${\vec{n}}_{i}$, Figure 2, where *r *was denoted as 'real' and *i *- as 'imaginary"). The real imaged circle normal vectors from the left (*l*) image ${\vec{n}}_{rl}$ and from the right (*r*) image ${\vec{n}}_{rr}$ coincided. The imaginary circle normal vectors ${\vec{n}}_{il}$ and ${\vec{n}}_{ir}$ did not coincide.

### Determination of the 3D position and scaling factor of the fiducial marker

To calculate the relative 3D position of the fiducial marker to the camera, the perspective projection of the four black circles centres (*r*_*i *_in Eq. 2, 1 ≤ *i *≤ 4) of the fiducial marker, called from now on as the fiducial quadrangle, had to be converted into orthogonal projections on the same image-plane (*p*_*i *_in Eq. 3). It was assumed that the image-plane passed through the cross point of the two diagonals of the fiducial quadrangle.

(2) $$r_{i}=E+t_{i}{\vec{e}}_{i},$$

(3) $$p_{i}=E+h_{i}{\vec{e}}_{i},$$

where *E *is the principal point of the image, 1 ≤ *i *≤ 4, $\vec{e}$ is the unit vector, *t *and *h *are the lengths of the corresponding vectors. The four orthogonal projection points of the fiducial quadrangle tops (*p*_*i*_) lay on the rays from *E *to *r*_*i*_. The cross point divided the diagonals of the fiducial quadrangle under known length ratios used to calculate the *p*_*i*_.

Let denote *M*_*0*_(x_*0i*_,y_*0i,*_z_*0i*_) as the known coordinates of the fiducial quadrangle with the coordinates origin in the diagonals cross point. The orthogonal projection of *M*_*0 *_onto the image-plane can be described as *M*_1_(x_1__*i*_,y_1__*i,*_z_1__*i*_):

(4) $$M_{1}=kRM_{0}+t,$$

Where *k *is a scaling factor, *R *is a rotation matrix and *t(x*_*t*_*,y*_*t*_*,z*_*t*_) is a translation vector. The *z*-components of *M*_*1 *_were unknown. Therefore the equation (4) was simplified for the known *x *and *y *components and the following equation was derived:

(5) s=A\q,

where $A=\left[\begin{array}{cccc} x_{01} & y_{01} & 0 & 0\\ 0 & 0 & x_{01} & y_{01}\\ . & & & \\ . & & & \\ . & & & \\ x_{04} & y_{04} & 0 & 0\\ 0 & 0 & x_{04} & y_{04} \end{array}\right]$, $q=\left[\begin{array}{c} x_{11}-x_{t}\\ y_{11}-y_{t}\\ .\\ .\\ .\\ x_{14}-x_{t}\\ y_{14}-y_{t} \end{array}\right]$ and "\" is backslash or matrix left division. If *A *were a square matrix, *A\q *would be roughly the same as *A*^*-1*^*q*. But in our case *A *is an *m*-by-*n *(*m *= 8 and *n *= 4) matrix with *m *≠ *n *and *q *is a column vector with *m *components, then *s = A\q *is the solution in the least squares sense to the under- or over-determined system of equations *As = q*.

In order to calculate the scaling factor *k*, the elements of the vector *s *were rearranged into a 2 by 2 matrix *S*. After the singular value decomposition (*svd*) [15,24] of matrix *S *the largest singular value of *S *was equal to the scaling factor *k*:

(6) k=max(svd(S))

Moreover, the four elements of the matrix *S *divided by the factor *k *are the four elements of the matrix *R*:

$$R=\left[\begin{array}{ccc} c_{11} & c_{12} & c_{13}\\ c_{21} & c_{22} & c_{23}\\ c_{31} & c_{32} & c_{33} \end{array}\right],\text{ where }s/k=\left[\begin{array}{c} c_{11}\\ c_{12}\\ c_{21}\\ c_{22} \end{array}\right]\text{ and }S/k=\left[\begin{array}{cc} c_{11} & c_{12}\\ c_{21} & c_{22} \end{array}\right].$$

In order to reconstruct the matrix *R*, the property of a unit matrix was used where the sum of the squared column or row elements is equal to one. The signs in the third column and row of *R *were reconstructed by means of another property where determinant of *R *is equal to one. Now only two right orthogonal matrixes remained which corresponded to the two possible 3D positions of the fiducial quadrangle. In order to choose the matrix *R *which described the real imaged position of the fiducial quadrangle, the property of the perspective projection to converge to the perspective projection centre *O(0,0,0) *was used. The lines had to converge to *O *when they passed through the four points of the fiducial marker *p*_*i*_*(x*_*pi*_*,y*_*pi*_*,z*_*pi*_) and their perspective projection *r*_*i*_*(x*_*ri*_*,y*_*ri*_*,d)*, where *d *was the distance from *O *to the image *XY*-plane and the coordinates of *p*_*i *_were calculated as in the equation (4).

### 3D reconstruction of markers

The 3D position of the fiducial marker regarding the camera coordinate system was determined by means of the described above rotation matrix *R*, the scaling factor *k *and the translation vector *t*. The two cameras were used to simultaneously acquire two images of the same scene. After rigid body transformation of $O_{j}^{c}$ and the perspective projections of markers $m_{jn}^{c}$ into the fiducial marker coordinate system, the 3D positions of markers with respect to the fiducial marker were reconstructed. The single 3D marker position $m_{n}^{f}$ was the estimated intersection point of the two rays from the perspective projection centre $O_{j}^{f}$ to the corresponding marker perspective projection $m_{jn}^{f}$[25], where 1 ≤ *j *≤ 2, *n *was an integer between 1 and the number of markers, *c *was denoted as "camera coordinate system" and *f *was denoted as "fiducial marker coordinate system".

### Evaluation experiments

#### Experiment 1: Inter-marker distance measurement

In order to evaluate the accuracy (agreement between the measured and reference values) and precision (closeness of measurement values to each other under similar experimental conditions [26]) of the presented measurement system an 8 × 8 grid of black circles was printed on a white surface and adhered to a 10 × 10 cm plate. The test distance between adjacent circles centres in horizontal and vertical directions amounted to 10 mm. Two grids with circles of 1 and 2 mm diameter were prepared to perform the following tests:

##### Test A - Translating Camera

**Figure 3:**
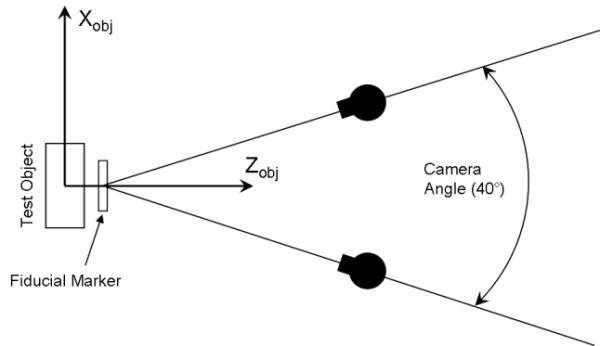
**Top view of the camera setup**. The *Y*_*obj *_axis is directed towards the reader.

The plate with the grid of circles was initially captured in close proximity and above the fiducial marker. The test object *Z*_*obj *_axis was perpendicular to the plate surface and parallel to the pin between the cameras. *X*_*obj *_axis was directed horizontally and *Y*_*obj *_axis - vertically (Figure 3). After that, the cameras were moved seven times farther away from the circles grid in step of approximately 1 cm along the *Z*_*obj *_axis while in each camera position the scene was captured. The test was repeated five times.

##### Test B - Rotating Plate

The grid of circles was captured eight times after the plate was rotated about *X*_*obj *_axis. The rotation angles were approximately -60, -45, -30, -15, 15, 30, 45 and 60°. The test was repeated five times.

##### Test C - Rotating Cameras

Similar as in the test B but here the cameras were rotated about *Y*_*obj *_axis. The rotation angles were approximately -60, -45, -30, -15, 15, 30, 45 and 60°. The test was repeated five times.

### Precision test of image processing

The image processing precision was determined by examining the variation of the 112 measured distances between adjacent circles centres in horizontal and vertical directions during the image processing. The 8 × 8 grid of circles was captured in five different positions. Then every of five image-pairs was processed 10 times. The captured positions were: one from the test A (the initial position), two from the test B (-30 and 30° rotation about *X*_*obj*_) and two from the test C (-30 and 30° rotation about *Y*_*obj*_). The variation in distances was calculated by means of the Root Mean Squares error as shown in the following equation:

(7) $$\varepsilon =\sqrt{\frac{1}{l\times m\times n}\sum_{k=1}^{l=5}\sum_{i=1}^{m=112}\sum_{j=1}^{n=10}{\left(d_{kij}-{\bar{d}}_{ki}\right)}^{2}},$$

where ${\bar{d}}_{ki}$ is the mean value of the *i*'s inter-marker distance on the grid of circles in the *k*'s position measured 10 times (*d*_*kij*_).

### System repeatability test

In this test the influence of the system rebooting on the 112 measured distances between adjacent circles centres in horizontal and vertical directions was examined. A single position of the grid of circles from the test A (the initial position) was captured 10 times. After acquisition of each capture the measurement system was shut down and started again. Every time, the zoom and focus had to be adjusted to the default values used during the pre-calibration. The variation in distances was calculated similarly to the image processing precision test by means of the Root Mean Squares error (7).

### Precision test on cadaveric specimen

**Figure 4:**
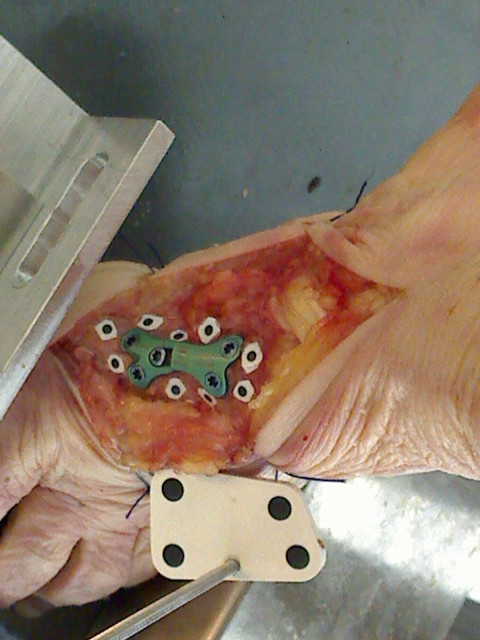
**Marker test on the cadaveric foot specimen**. The single right camera picture. The nine markers were attached on the first metatarsal bone fragments fixing with a locking screw plate after a proximal osteotomy. The fiducial marker was placed in the middle of the lower image part.

In order to test how the background colours of the connective tissue influence the precision of marker detection, a human foot specimen was used. The foot was fresh frozen and stored in a plastic bag at -20°C. The specimen (left, female, 79 years old, without degenerative changes of the connective tissue) was thawed at room temperature for 24 hours. Then the first metatarsal bone was prepared free of the surrounding tissue, proximally osteotomized and fixed with a locking screw plate. Nine markers were attached by means of acrylic superglue on the parts of the first metatarsal bone. The markers were 2 mm black circles on a white water resistant background (0.5 mm thin pieces approximately of a 4 mm diameter, Figure 4).

Five different pictures of the specimen were acquired by means of the dual cameras and then each was processed 10 times. The variation in all possible distances between the nine markers ($m=C_{9}^{2}=36$) was calculated similarly to the image processing precision test by means of the Root Mean Squares error (7).

#### Experiment 2: marker displacement measurement

In this experiment two plastic plates were used to determine the translational and rotational accuracy and precision when one plate was moved in respect to another static plate. Each plate was equipped with four black circles (mounting accuracy 0.005 mm). The circles were on the tops of a square whose side length amounted to15 mm. Two plate types were manufactured for circle sizes of 1 and 2 mm diameter.

##### Rotation test

In this test the *Z*_*obj *_axis of the stationary plate was perpendicular to the plate surface and parallel to the pin between the cameras. In the zero position the non-stationary plate was coplanar to the stationary plate and the circles on the corresponded square sides were collinear. The non-stationary plate was rotated about *X*_*obj *_, *Y*_*obj *_and *Z*_*obj *_axes by means of a headpiece on a milling unit (Deckel Maho Pfronten GmbH, Germany) used for precise rotation with a step of 2.25 ± 0.025° trough the range of 90° (± 45° from the zero position).

The magnitude of rotation between the stationary and non-stationary plates was calculated by means of a detection of the rotation matrix describing the rotation between the plates [27]. From the rotation matrix was calculated "the attitude vector" $\bar{a}=\bar{n}\alpha$[28], where $\bar{n}$ is the unit vector about which the scalar rotation *α *occurs. Then "the attitude vector" was orthogonally decomposed onto the *X*_*obj *_, *Y*_*obj *_and *Z*_*obj *_axes [29] of the stationary plate.

##### Translation test

In order to determine the accuracy and precision of translational measurements the non-stationary plate was translated from the zero position along each of the *X*_*obj*_, *Y*_*obj *_and *Z*_*obj *_axes in amount of 0.5, 1 and 5 mm. The translations were performed by means of the translational manipulator (ThorLabs Inc. Europe, Karlsfeld, Germany). Its accuracy (0.005 mm) and precision (± 0.002 mm) was characterized using laser interferometry in the previous study [30]. The test was repeated five times.

### Statistical analysis

The error value was calculated as a difference between the reference value and the measured value. All collected error values were examined with Jarque Bera test to verify that the data was normally distributed. Regarding this test, many of the error values sets were significant (p < 0.05) non normally distributed. Therefore non parametric statistical methods were applied. The accuracy of the presented measurement system was represented by means of the mean and median error [31]. The precision was calculated as a standard deviation [31] or as Root Mean Squares error (7).

The median error was presented due to the applied non parametric methods. The comparison of medians was performed by means of Kruskal Wallis test. The variance of the error values was compared by means of the Levene's test. All statistics were performed using MATLAB (The MathWorks Inc., Natick, MA, USA). The values of significant difference or significant sameness [32] were set at p < 0.05 and p > 0.95 respectively.

## Results

### Experiment 1: detection of Inter-marker distance

#### Tests A, B and C - 10 mm distance detection

Results in this experiment section for the accuracy and precision were excellent for both marker sizes (Table 1). The total accuracy and precision (mean (median) ± standard deviation) amounted for 1 mm markers 0.009 (-0.002) ± 0.1002 mm and for 2 mm markers 0.008 (-0.004) ± 0.1003 mm. Levene's test showed that the variances of 1 mm and 2 mm markers during the tests A, B and C were significantly the same (p = 0.956). For both marker sizes, variance of distance detection errors occurred during the test B were significantly higher (p < 0.001 for 1 mm and p = 0.002 for 2 mm markers) as during the test C and A. Kruskal Wallis tests showed that the medians for 2 mm markers were significantly different (p < 0.001) between the tests A, B and C. The medians for 1 mm markers were significantly different (p < 0.001) between the test A and B, otherwise the p values were very small (0.05 < p < 0.08).

**Table 1:** Detection of the 10 mm inter marker distance

Marker	**Test A error (*Z***_***obj***_**)**	**Test B error (*X***_***obj***_**)**	**Test C error (*Y***_***obj***_**)**
ø1 mm	0.011 (0.002) ± 0.094	0.009 (-0.007) ± 0.110	0.007 (-0.002) ± 0.096

ø2 mm	0.023 (0.013) ± 0.098	0.007 (-0.014) ± 0.103	-0.006 (-0.02) ± 0.098

Results of the measurement system accuracy and precision evaluation represented by mean (median) error ± standard deviation in mm. In order to show about or along which axis the test was performed the test object axes were indicated in the parentheses in the first row.

#### Image processing precision and repeatability tests

The image processing precision test revealed the Root Mean Squares error for 1 and 2 mm Markers at the level of 0.0044 and 0.0051 mm, respectively. Levene's multiple-sample test showed a significantly (p < 0.001) higher variance of mean distance deviation for 2 mm Markers in comparison to 1 mm.

The system repeatability test detected the Root Mean Squares error for both marker sizes at the level of 0.013 mm. Levene's test showed that the variance of the distance detection errors for both marker sizes was significant (p = 0.98) the same.

The image processing precision test on the foot specimen showed the Root Mean Squares error at the level of 0.0035 mm.

### Experiment 2: measurement of displacement

#### Rotation test

Results in this experiment section for the accuracy and precision were very good for both marker sizes (Table 2). The total accuracy and precision (mean (median) ± standard deviation) amounted for 1 mm markers 0.054 (0.027) ± 0.190° and for 2 mm markers 0.062 (0.028) ± 0.154°. No significant differences in variance (p = 0.259) between 1 and 2 mm markers were observed regarding the Levene's test. Regarding Kruskal Wallis test the accuracy of the rotation about the *Z*_*obj *_was significant better as about *X*_*obj *_and *Y*_*obj *_axes with p = 0.004 and p < 0.001 for 1 and 2 mm markers respectively. The better precision of the rotation about the *Z*_*obj *_showed also Levene's test with p = 0.012 and p < 0.001 for 1 and 2 mm markers respectively.

**Table 2:** Rotation test

Marker	**Rotation error *X***_***obj***_	**Rotation error *Y***_***obj***_	**Rotation error *Z***_***obj***_
ø1 mm	0.08 (0.08) ± 0.159	0.082 (0.123) ± 0.281	0.001 (-0.006) ± 0.035
ø2 mm	0.105(0.092) ± 0.160	0.080 (0.098) ± 0.192	0.000 (-0.008) ± 0.062

Results of the measurement system evaluation: accuracy and precision represented by mean (median) error ± standard deviation in degree.

#### Translation test

The stereophotogrammetrical system delivered very good results for translational accuracy and precision (Table 3). The total accuracy and precision (mean (median) ± standard deviation) across the translational test were 0.008 (0.005) ± 0.053 mm for 1 mm markers and -0.001 (-0.002) ± 0.038 mm for 2 mm markers. The Levene's test revealed significantly (p = 0.002) higher variance of the measurements errors with 1 mm markers versus 2 mm markers. The variance of 5 mm translation errors for both marker sizes was significantly higher (p = 0.0004) than the error variance of the smaller 0.5 and 1 mm translations. The error variance of the translations along the *Z*_*obj *_axis was significantly higher (p < 0.001) as along the *X*_*obj *_and *Y*_*obj *_axes.

**Table 3:** Translation test

Marker	Transl. mm	**Translation error *X***_***obj***_	**Translation error *Y***_***obj***_	**Translation error *Z***_***obj***_
	0.5	-0.001(-0.003) ± 0.014	0.024(0.019) ± 0.031	0.009(0.006) ± 0.058
ø1 mm	1	-0.006(-0.007) ± 0.017	0.033(0.037) ± 0.030	-0.011(0.001) ± 0.062
	5	-0.027(-0.026) ± 0.014	0.032(0.046) ± 0.091	0.021(0.023) ± 0.065

	0.5	-0.002(-0.006) ± 0.016	0.014(0.011) ± 0.021	-0.019(-0.026) ± 0.032
ø2 mm	1	-0.003(-0.004) ± 0.012	0.008(0.01) ± 0.013	0.003(-0.0003) ± 0.057
	5	-0.045(-0.045) ± 0.011	-0.013(-0.016) ± 0.034	0.052(0.046) ± 0.041

Results of the measurement system evaluation: accuracy and precision represented by mean (median) error ± standard deviation in mm.

## Discussion

This study demonstrated that the 3D stereophotogrammetrical system based on the tracking of flat round markers can accurately measure the distances and movements within a small measurement volume. The algorithms to detect the centre of the projected circle and to estimate the camera pose using the fiducial marker were presented. The evaluation tests were designed in order to test the measurement accuracy and precision of distances and movements expected during the tracking of markers fixed on small bones or ligaments. Despite the fact that the presented system processes only static images, in comparison to similar systems using proprietary vendor-specific hardware, the presented system is a very small fraction of the cost. Future goals include the possibility of making the presented measurement system able to extract the tracked markers from a video stream.

The developed algorithm of the detection of the projected circle centre functioned with a single projected circle while other algorithms require for the detection for example concentric circles [33] or coplanar circles [18,20,21]. On the other hand, the presented algorithm of the projected circle centre detection required the principal point and principal distance to be known from the pre-calibration. The quality of the pre-calibration could play a determinant role in the accuracy and precision [34] of the presented measurement system, when the measurement conditions were optimal. This played an important role during the camera pose estimation using the fiducial marker where the pose estimation was optimized by means of the direct transformation of the fiducial marker perspective projection into its orthogonal projection, the least squares algorithm using to solve the equation 5 and the reconstruction of the rotation matrix using the orthogonal matrix constrains.

The remaining inaccuracies of the camera pre-calibration could explain the reduction of precision when lager distances were measured. This could be corroborated by the smaller image processing precision (± 0.005 mm) in comparison to the precision ranged from ± 0.011 to ± 0.11 mm while the distances and movements were measured. The system repeatability (± 0.013 mm) also influenced the distance and movement measurements precision because of small discrepancies occurred during the system adjusting the zoom and focus values. Lujan et al. also reported that "lager translations/rotations reduced kinematic accuracy" [5].

In the presented study, the precision was calculated as the standard deviation or as the Root Mean Squares error (equation 7) because of the calculation similarity. Therefore these two parameters had to be more comparable than the mean standard deviation [6] or the two standard deviations [5], which were used instead of the Root Mean Squares error in the related studies [5,6].

The accuracy values were very close to zero and ranged from -0.045 to 0.052 mm for translations and from -0.008 to 0.105° for rotations. The presented system showed comparable results in accuracy for the similar displacements measurements in the study of Lujan et al. (0.034 mm for the translations and 0.132° for the rotations) [5] and in the study of Everaert et al. the accuracy ranged from 0 to 0.05 mm [6].

The measurement with the flat round markers was limited by the angle *λ *between the normal vector to the marker plane and the camera view axis (0°≤*λ<90°*). When this angle became too close to 90°, there were difficulties to fit the ellipse of the projected circle because the ellipse became too slim. Therefore the axes ratio of the ellipse could be a criterion of the critical value of the angle *λ. *This axes ratio criterion was set at 0.2 (correspond approximately to *λ *= 78.5°). Therefore the angle range between the *Z*_*obj *_and the pin with the fiducial marker, what was mounted between the cameras, was set at ± 60° (Figure 1 and 3, test B and C).

It has been stated that: "The accuracy of the target location deteriorates if the number of edge pixels compared to central pixels increases, because of the uncertain grey values of the edge" [19]. This phenomenon occurred when the marker size became smaller [5] and the angle *λ *for the flat round markers, bigger. Due to using the zoom and good camera resolution, the marker size of 1 or 2 mm diameter showed in the tests A, B and C the significant agreement in precision. "Zoom lenses are used extensively in computer vision to overcome the limited resolution" [35]. Both cameras of the presented system were calibrated for the fixed zoom and focus settings because Wiley and Wong admitted in their study that: "There were significant changes in the distortion characteristics with changes in the focal setting. However, the pattern of change for a given camera-lens combination was very systematic and stable over time" [35], what was confirmed through the good precision values (± 0.013 mm) of the repeatability test.

The black circle on the white background - this is advantageous for the edge detection colour combination remained during the test on the cadaveric foot specimen. Therefore the colours of connective tissues surrounding the markers did not deteriorate the precision of the presented measurement system. Nevertheless the use of flat round retroreflective markers may be more advantageous because the maker size reduction.

The measurement of the displacements in the second experiment showed typical distribution of the measurement errors for this camera setup [5,31]. Regarding this distribution the presented measurement system performed the best translation measurement in the *XY*_*obj*_-plane and the best rotation measurement, when rotations occurred about the *Z*_*obj *_axes.

## Conclusions

The study demonstrated that the handy 3D stereophotogrammetrical system based on the tracking of the flat round markers within a small measurement volume with respect to the fiducial marker can accurately measure the distances and movements. The evaluation experiments of the 10 mm distances measurement showed the total accuracy of 0.0086 mm (mean error) and the precision of ± 0.1002 mm (standard deviation). The translations from 0.5 mm to 5 mm were measured with the total accuracy of 0.0038 mm and the precision of ± 0.0461 mm. The rotations of 2.25° amount were measured with the entire accuracy of 0.058° and the precision of ± 0.172°. These levels of accuracy and precision may provide opportunities for new applications of stereophotogrammetrical analysis in orthopaedic research projects, focusing on small displacements in a small measurement volume.

## Competing interests

The authors declare that they have no competing interests.

## Authors' contributions

EB developed the measurement system design and the algorithms, written the manuscript, carried out the evaluation study and analyzed the data. GO and CP were involved in the drafting and revision of the manuscript. CH contributed to the evaluation tests design and was involved in the drafting and revision of the manuscript. HW contributed to the evaluation test design and performance. HA was involved in the study design and the performance of the evaluation test. CS was involved in the study design. All authors read and approved the final version of the manuscript.
